# Highly Efficient Site-Specific Mutagenesis in Malaria Mosquitoes Using CRISPR

**DOI:** 10.1534/g3.117.1134

**Published:** 2017-12-11

**Authors:** Ming Li, Omar S. Akbari, Bradley J. White

**Affiliations:** Department of Entomology, University of California, Riverside, California 92521

**Keywords:** *Anopheles*, gene drive, reverse genetics, transgenics, CRISPR, Cas9

## Abstract

*Anopheles* mosquitoes transmit at least 200 million annual malaria infections worldwide. Despite considerable genomic resources, mechanistic understanding of biological processes in *Anopheles* has been hampered by a lack of tools for reverse genetics. Here, we report successful application of the CRISPR/Cas9 system for highly efficient, site-specific mutagenesis in the diverse malaria vectors *Anopheles albimanus*, *A. coluzzii*, and *A. funestus*. When guide RNAs (gRNAs) and Cas9 protein are injected at high concentration, germline mutations are common and usually biallelic, allowing for the rapid creation of stable mutant lines for reverse genetic analysis. Our protocol should enable researchers to dissect the molecular and cellular basis of anopheline traits critical to successful disease transmission, potentially exposing new targets for malaria control.

*Anopheles* mosquitoes are the exclusive vectors of mammalian malaria (White *et al.* 2011). Over the past decade, human malaria deaths have declined by nearly 50%, primarily due to increased use of insecticides that target the mosquito vector (Bhatt *et al.* 2015). However, emerging physiological and behavioral resistance in *Anopheles* populations threatens the sustainability of insecticidal control (David *et al.* 2005; Edi *et al.* 2014; Sougoufara *et al.* 2014; Ranson and Lissenden 2016). In order to maintain and extend the hard-won progress of the past decade, novel vector control strategies need to be developed and combined with traditional chemical control. The development of new tools in the fight against malaria mosquitoes is contingent upon improved mechanistic knowledge of myriad mosquito biological processing, including blood feeding, gametogenesis, gustation, immunity, olfaction, and metabolism, among many others.

In 2002, the African malaria mosquito *Anopheles gambiae* was the second arthropod to have its genome sequenced and, more recently, the genomes of 16 other anophelines were sequenced (Holt *et al.* 2002; Neafsey *et al.* 2013). Despite considerable genomic resources, progress in dissecting the molecular and cellular biology of malaria mosquitoes has been slow, primarily due to the difficulty in performing reverse genetic techniques that are routine in model organisms. Currently, the vast majority of *Anopheles* genes have no known function (Giraldo-Calderón *et al.* 2015), impeding the development of novel vector control strategies reliant upon understanding how individual genes contribute to the biology of the mosquito. Previously, genome editing in *Anopheles* relied on either transposon-based transgenesis with no control over where an insertion occurred (Grossman *et al.* 2001; Nolan *et al.* 2002; Perera *et al.* 2002; Meredith *et al.* 2011; Carballar-Lejarazú *et al.* 2013; Pondeville *et al.* 2014) or highly inefficient and expensive, site-specific genome editing technologies such as zinc finger nucleases or transcription activator-like effector nucleases (Windbichler *et al.* 2007; Smidler *et al.* 2013). Recently, the CRISPR/Cas9 genome editing technique has been successfully applied to a diversity of organisms (Bassett *et al.* 2013; Hsu *et al.* 2014; Port *et al.* 2014; Basu *et al.* 2015; Dong *et al.* 2015; Gantz *et al.* 2015; Hall *et al.* 2015; Kistler *et al.* 2015; Barrangou and Doudna 2016; Hammond *et al.* 2016; Li *et al.* 2017; Sharma *et al.* 2017; Staahl *et al.* 2017; Li *et al.* 2017a; Li *et al.* 2017b). With this technology, researchers can directly edit or modulate DNA sequences, allowing them to study the function of genes *in vivo* (Hsu *et al.* 2014). When used for site-directed mutagenesis, Cas9 protein and a small gRNA (sgRNA) that is complementary to a target sequence in the genome are delivered to germ cells. The Cas9 and sgRNA complex bind to the target sequence and cause a double-strand break, which will be repaired through nonhomologous end joining (NHEJ) or microhomology-mediated end joining (MMEJ) resulting in mismatches and indels relative to wild-type sequence (Bae *et al.* 2014; Basu *et al.* 2015; Maruyama *et al.* 2015). When exons are targeted, such mutagenesis will often result in premature stop codons or frameshifts that disrupt protein function. Despite high mutagenesis efficiency in other organisms, it is unclear if the CRISPR/Cas9 system will prove to be efficient in *Anopheles*, as egg injection alone often results in extremely high mortality and low transformation efficiencies, perhaps due to the inherent fragility of the eggs themselves. Here, we report successful development of an efficient site-specific mutagenesis protocol using the CRISPR/Cas9 system in various anophelines, facilitating reverse genetics in this important group of disease vectors.

## Materials and Methods

### Mosquito strains

Four mosquito colonies were used in this study: *A. coluzzii* wild-type strain NGS, *A. gambiae* white-eyed mutant strain M2 (MRA-105), *A. albimanus* wild-type strain *STECLA* (MRA-126), and *A. funestus* wild-type strain *FUMOZ* (MRA-127). Strains with accession numbers were obtained from the Malaria Research and Reference Reagent Resource Center (MR4). Mosquitoes were maintained in insectaries at the University of California, Riverside (UCR), under standard conditions (White *et al.* 2011).

### sgRNA design and generation

gRNAs were designed by searching both the sense and antisense strand of exon 2 of the *white* gene (ACOM037804, AALB006905, and AFUN003538) for the presence of protospacer-adjacent motifs with the sequence NGG using ZIFIT (http://zifit.partners.org/ZiFiT/ChoiceMenu.aspx) and CRISPR Design (http://crispr.mit.edu/) (Xie *et al.* 2014). Linear, double-stranded DNA templates for sgRNAs were generated by performing template-free PCR with Q5 high-fidelity DNA polymerase (NEB), the forward primer of each gRNA, and universal-sgRNAR. PCR conditions included an initial denaturation step of 98° for 30 sec, followed by 35 cycles of 98° for 10 sec, 58° for 10 sec, and 72° for 10 sec, followed by a final extension at 72° for 2 min. PCR products were purified with magnetic beads using standard protocols. gRNAs were generated by *in vitro* transcription (AM1334; Life Technologies) using 300 ng purified DNA as template in an overnight reaction incubated at 37°. MegaClear columns (AM1908; Life Technologies) were used to purify sgRNAs, which were then diluted to 1 μg/μl, aliquoted, and stored at 80° until use. Three possible off-target sites of each sgRNA in the different mosquito species were identified based on the CHOPCHOPv2 software (Labun *et al.* 2016) and local sgRNACas9 package (Xie *et al.* 2014), and analyzed by using a T7 endonuclease I (T7EI) assay, respectively. Briefly, genomic DNA was extracted from mosquitoes with the DNeasy blood & tissue kit (QIAGEN) following the manufacturer’s protocol. Target loci were amplified by PCR and PCR product was purified with a MinElute PCR purification Kit (QIAGEN). Next, 2 μl of NEB buffer 2, 200 ng of purified PCR product, and ddH_2_O (to a total volume of 19 μl) were mixed together and a hybridization reaction conducted in a PCR cycler with 5 min, 95°; ramp down to 85° at −2°/sec; ramp down to 25° at −0.1°/sec; and hold at 4°. Next, 1 μl (10 U) of T7EI was added and the reaction incubated at 37° for 15 min. The reaction was stopped by adding 2 μl of 0.25 M EDTA and loaded immediately on a 1.5% agarose gel. All primer sequences are listed in Supplemental Material, Table S1 in File S1. Recombinant Cas9 protein from *Streptococcus pyogenes* was purchased from PNA Bio (CP01) and diluted to 1 μg/μl in nuclease-free water with 20% glycerol, and stored in aliquots at −80°.

### Microinjection

Mixed sex pupae were allowed to eclose into a single (L24.5 × W24.5 × H24.5 cm) cage. After allowing 5 d for mating, females were offered a bovine bloodmeal using the Hemotek (model# PS5) blood feeding system. A minimum of 60 hr was allowed for oogenesis, after which ovicups filled with ddH_2_0 and lined with filter paper were introduced into cages, and females were allowed to oviposit in the dark. After ∼15 min, the ovicup was removed and unmelanized eggs were transferred onto a glass slide and rapidly aligned against a wet piece of filter paper. Aluminosilicate needles (AF100-64-10; Sutter) pulled on a Sutter P-1000 needle puller (heat 605, velocity 130, delay 80, pull 70, and pressure 500) and beveled using a Sutter BV-10 beveler were used for injections. An Eppendorf Femtojet was used to power injections, which were performed under a compound microscope at 100× magnification. Since eggs were injected prior to melanization, only 10–20 eggs were injected at a time, after which fresh eggs were obtained. After injection, eggs were floated in ddH_2_0 and allowed to hatch spontaneously.

### Mutation screens

The white-eye phenotype of G0 and G1 mosquitoes was assessed and photographed under a Leica M165 FC stereomicroscope. To molecularly characterize CRISPR/Cas9-induced mutations, genomic DNA was extracted from a single mosquito with a DNeasy blood & tissue kit (QIAGEN) and target loci were amplified by PCR. For T7EI assays, 1 μl of T7EI (NEB) was added to 19 μl of PCR product, digested for 15 min at 37°, and visualized on a 2% agarose electrophoresis gel stained with ethidium bromide. To characterize mutations introduced during NHEJ or MMEJ, PCR products containing the sgRNA target site were amplified, cloned into TOPO TA vectors (Life Technologies), purified, and Sanger sequenced at the UCR Genomics core.

### Data availability

Genomic DNA from mosquito strains produced here will be made available upon request.

## Results

In order to rapidly and easily detect successful CRISPR/Cas9 mutagenesis, we wanted to target a gene where knockout of only a single allele produces a visible phenotype. However, no dominant visible mutations for *Anopheles* have been previously reported. Thus, we chose to target the *white* gene, which codes a protein critical for eye pigment transport (Besansky *et al.* 1995). Knockout of the white gene results in a change from wild-type red eye color to white (unpigmented) eye color, a simple phenotype to score. Although the white allele is recessive, it is located on the X chromosome and thus hemizygous in male anophelines (XY sex determination system), meaning that successful knockout of a single allele in males will result in the white-eye phenotype.

### Mutagenesis efficiency is concentration- and sgRNA-dependent

**Figure 1:**
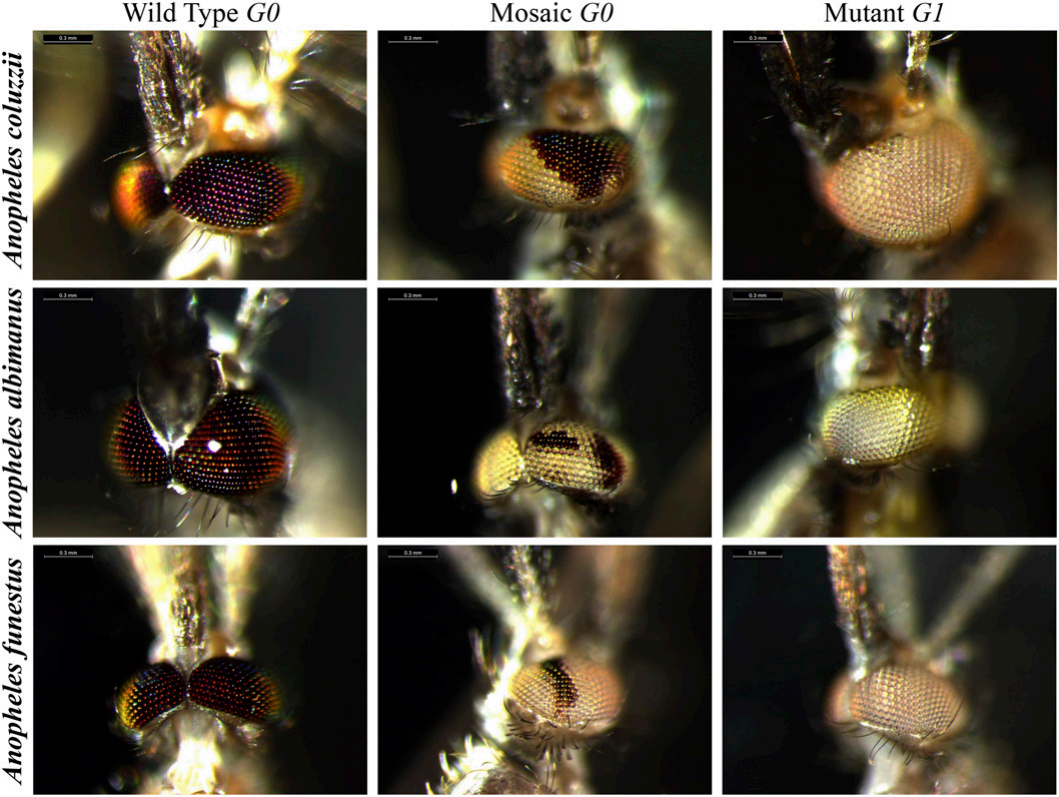
CRISPR/Cas9 efficiently generates heritable, site-specific mutations in diverse *Anopheles* mosquitoes. On the left, representative images of wild-type anopheline eyes are shown for each species. In the center are representative G_0_ mosaic white-eyed mutant mosquitoes that were injected with sgRNA and Cas9 as embryos. On the right are representative homozygous white-eyed mutant G_1_ mosquitoes generated by crossing mosaic G_0_ male and female mosquitoes. CRISPR, clustered regularly interspaced short palindromic repeats; sgRNA, small guide RNA.

*A. coluzzii* belongs to the *A. gambiae* complex, which includes a number of major African malaria vectors. To determine the efficacy of CRISPR/Cas9 mutagenesis in this species complex, we designed two sgRNAs targeting exon 2 of the *white protein* gene (ACOM037804). First, we used AcsgRNA1 to test how different concentrations of both the sgRNA and Cas9 protein affected mutagenesis rates. We found that both embryo survival and mutagenesis rate were sgRNA and Cas9 concentration-dependent (Table 1). Greater than 50% of embryos survived control injections with only water; however, survival rates for embryos (37%) injected with even the lowest concentration of sgRNA and Cas9 decreased relative to control. With increasing concentrations of sgRNA and Cas9, embryo survival further decreased. Indeed, only 11% of embryos survived injections with the highest concentrations tested. Conversely, concentrations of sgRNA and Cas9 were positively correlated with mutagenesis rates; 46% of males injected with the lowest concentration had mosaic white eyes, while a remarkable 100% of males injected with the highest concentration had mosaic eyes (Figure 1 and Table 1). Importantly, at higher injection concentrations, a majority of injected *females* also had mosaic eyes. Since the white gene is recessive, the production of mosaic females demonstrates that the CRISPR/Cas9 system can mutate both copies of diploid *Anopheles* genes. Notably, we also observed G0-injected males and females with completely white eyes, suggesting that the vast majority of cells in the eyes were mutated. Based on the above results, we used an sgRNA concentration of 120 ng/μl and a Cas9 protein concentration of 300 ng/μl, which balances survival and mutagenesis efficiency, to further explore the CRISPR/Cas9 system in *Anopheles*. To determine if the sgRNA sequence had an effect on mutagenesis rate, we compared AcsgRNA1 from above against a second sgRNA (AcsgRNA2) targeting white. We found that AcsgRNA1 (93, 87%) produced mosaic G0 males and females at a much higher frequency than AcsgRNA2 (32, 25%), suggesting that the sgRNA sequence can have a large impact on mutagenesis efficiency (Table 2).

**Table 1:** Effect of sgRNA and Cas9 concentration on *A. coluzzii* survival and mutagenesis

AcsgRNA 1	Cas9	Number Injected	Survivors			Mosaic (%)	
			M	F	Total (%)	M (%)	F (%)
No injection	No injection	300	137	118	255 (85)	0	0
Water	Water	217	69	52	121 (56)	0	0
30 (ng/μl)	100 (ng/μl)	185	31	38	69 (37)	32 (46)	0
60	200	251	48	33	81 (32)	48 (59)	0
120	300	219	31	16	47 (21)	29 (94)	12 (75)
240	400	177	22	11	33 (19)	20 (91)	9 (82)
480	500	228	12	12	24 (11)	12 (100)	10 (83)

M, male; F, female.

**Table 2:** G_0_ and G_1_ mutagenesis rates in three different *Anopheles* species.

	**sgRNA**	**# Inj.**	**Survivors**			**Mosaic (%)**		**G**_**0**_ **M × White F**		**White M × G**_**0**_ **F**		**G**_**0**_ **M × G**_**0**_ **F**	
			*M*	*F*	*Total (%)*	*M (%)*	*F (%)*	*G_1_ Mutant M (%)*	*G_1_ Mutant F (%)*	*G_1_ Mutant M (%)*	*G_1_ Mutant F (%)*	*G_1_ Mutant M (%)*	*G_1_ Mutant F (%)*
***Anopheles coluzzii***	**AcsgRNA1**	612	76	62	138 (23)	71 (93)	54 (87)	991 (93)	117 (91)	851 (91)	1232 (94)	881 (86)	939 (81)
	**AcsgRNA2**	447	53	36	89 (20)	17 (32)	9 (25)	1038 (88)	1273 (84)	751 (89)	882 (91)	751 (45)	846 (49)
***Anopheles albimanus***	**AasgRNA1**	573	81	58	139 (24)	74 (91)	43 (74)	N/A		N/A		1317 (60)	1577 (62)
	**AasgRNA2**	511	79	68	147 (29)	0	0	N/A		N/A		N/A	N/A
***Anopheles funestus***	**AfsgRNA1**	237	15	11	26 (11)	8 (53)	7 (64)	N/A		N/A		53 (65)	92 (71)
	**AfsgRNA2**	352	21	16	37 (11)	5 (24)	4 (25)	N/A		N/A		37 (51)	62 (61)

### Confirmation of germline mutations and site specificity

While mosaic G0 mosquitoes can be used for reverse genetics, the creation of stable, mutant lines permits more thorough investigation of gene function. Thus, we wanted to determine the proportion of G0 mosaic-eyed *A. coluzzi* that possessed germline mutations. To obtain the germline mutation rate, we crossed G0 mosaic-eyed males with females of an existing white-eye mutant line of *A. coluzzii* (M2) that was established > 20 yr ago (Figure S2A in File S1) (Mason 1967; Besansky *et al.* 1995; Benedict *et al.* 1996). Hemizygous male progeny of this cross will all have white eyes since they inherit a maternal mutated white gene; however, homozygous females will only have white eyes if they inherit a mutant allele from both parents. A remarkable 93% of G0 mosaic males injected with AcsgRNA1 produced G1 females with white eyes, while 88% of G0 AcsgRNA2 mosaic males passed on white-eye mutations to G1 female progeny (Table 2). To determine if female mosquitoes with mosaic eyes could also pass on the mutation, we performed a bulk cross of mosaic G0 males with mosaic G0 females and found that 83% (male 86% and female 81%) of the G1 progeny from AcsgRNA1-injected mosquitoes had fully white eyes, while 47% (male 45% and female 49%) of G1 progeny from AcsgRNA2-injected mosquitoes had fully white eyes (Table 2). We have now maintained multiple, white-eyed mutant lines in the laboratory for >15 generations, proving that the mutations introduced by CRISPR/Cas9 are highly stable. The combination of good G0 survival, biallelic mutation, and high germline transmission allows for the rapid creation of knockout *A. coluzzii* lines using CRISPR/Cas9.

To confirm that the mosaic eye phenotype was caused by loss-of-function of the *white* gene, we performed a T7EI assay on five randomly chosen G0 AcsgRNA1 and AcsgRNA2 male mosquitoes with mosaic eyes. In the T7EI assay, T7EI will cut when NHEJ or MMEJ of the CRISPR-induced double strand break introduces a SNP or indel relative to the wild-type allele, whereas no digestion will occur in mosquitoes with two wild-type alleles. As expected, PCR fragments of the *white* gene from mosaic-eyed males were consistently cut into small bands by T7EI, while no activity was observed in nonmosaic male mosquitoes (Figure S4, A–E in File S1), confirming that the white-eye phenotype is caused by disruption of the *white* coding sequence. To sample the spectrum of mutations introduced by NHEJ or MMEJ, we performed Sanger sequencing of PCR products containing the two sgRNA target sites in G1 mosquitoes with white eyes. The sequencing results confirmed the presence of indels that were induced by NHEJ or MMEJ (Figure S3, A and B and Table S2 in File S1) in all mutant mosquitoes, which ranged in size from 2 to 54 bp. Finally, we screened for off-target activity of both sgRNAs by T7EI assay. Across three potential off-target loci for both sgRNAs, no evidence of mutagenesis was detected in G0 mosaic males, indicating high specificity of the sgRNAs (Figure S5 in File S1).

### CRISPR/CAS9 activity in diverse Anopheles

To determine the applicability of the CRISPR/Cas9 system to diverse *Anopheles* species, we performed injections targeting *white* in *A. albimanus* (a minor vector of malaria on South America) and *A. funestus* (a major, understudied malaria vector in Africa) (Neafsey *et al.* 2013). For each species, we designed two sgRNAs targeting *white* and injected the individual sgRNAs (120 ng/μl) and Cas9 (300 ng/μl) directly into eggs.

As found in other studies, *A. albimanus* survived injections at a rate comparable to *A. coluzzii* (Perera *et al.* 2002). For AasgRNA1, we found that 91% of G0 males had mosaic white eyes, while 74% of G0 females were mosaics. Interestingly, injection of AasgRNA2 produced no mosquitoes with mosaic eyes, further reinforcing the impact of sgRNA choice on mutagenesis efficiency. Since no previously generated white-eyed line of *A. albimanus* was available, we bulk crossed AasgRNA1 G0 mosaic males and females to determine germline mutation rates (Table 2). Over 61% of the G1 progeny from the cross possessed fully white eyes, suggesting high germline mutagenesis efficiency in *A. albimanus*. As with *A. coluzzii*, T7EI assays consistently detected mutations in white in G0 mosaic males (Figure S4 in File S1) and sequencing showed indels in mosaic males ranging from 2 to 11 bp (Figure S3 in File S1). Additionally, no mutations were detected using T7EI assays at three potential off-target sites for AasgRNA1 (Figure S5 in File S1).

**Figure 2:**
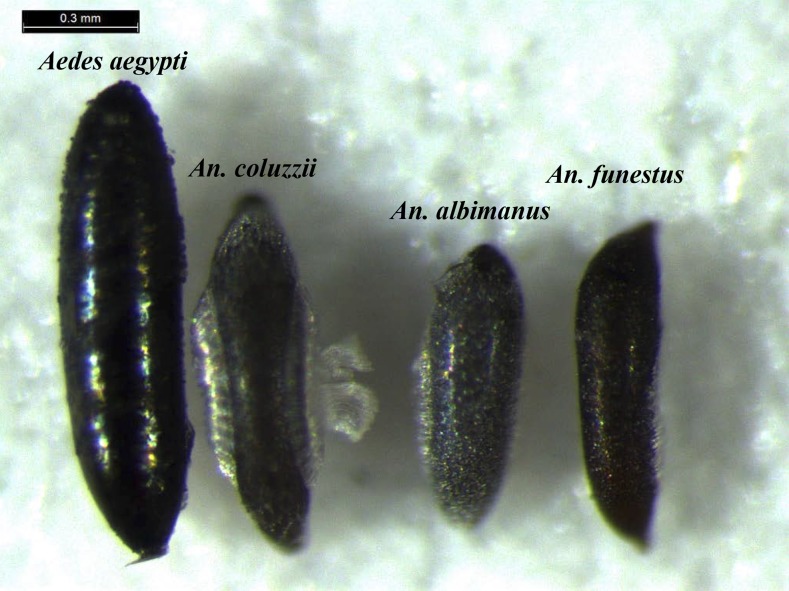
Morphology of eggs differs dramatically among anophelines. Eggs of the three species of *Anopheles* used in this study alongside an egg of the yellow fever mosquito *Ae. aegypti* for size comparison. Note the difference in pole shape between *A. albimanus* and *A. funestus* eggs, which likely contributes to differences in both survival and mutagenesis rates between these two species.

The survival rate of *A. funestus* embryos injected with either of two sgRNAs targeting the white gene (AfsgRNA1 and AfsgRNA2) was less than half that of *A. coluzzii* and *A. albimanus*. (Table 2). We attribute the lower survival rate to the unique morphology of *A. funestus* eggs at the poles (Figure 2), which makes injection challenging. Despite lower survival, a high proportion of G0 males (53 and 24%) and females (64 and 25%) for both sgRNAs displayed mosaic white eyes. Bulk crossing of G0 male and female mosaics (Figure S2B in File S1) produced 67% (AfsgRNA1) or 57% (AfsgRNA2) G1 progeny with fully white eyes, demonstrating high germline mutagenesis rates. As with previous species, the T7EI assay consistently identified mosaic males (Figure S4 in File S1) and Sanger sequencing revealed diverse indels (2–34 bp) in mutated males (Figure S3 in File S1). No mutagenic activity was detected for either sgRNA at the three most likely off-target genomic sites (Figure S5 in File S1).

## Discussion

The CRISPR/Cas9 system offers the possibility of precise, efficient, and cost-effective mutagenesis in nonmodel organisms (Barrangou and Doudna 2016). While considerable genomic resources have been developed for malaria mosquitoes, no efficient tools for performing reverse genetics in these species exist, slowing the development of genetics-based vector control. Studies of the CRISPR/Cas9 system in the distantly related (diverged 145–200 million years ago) mosquito *Aedes aegypti* have demonstrated G0 mutagenesis rates between 3 and 50%, with high variability among injection operators and different sgRNAs (Basu *et al.* 2015; Kistler *et al.* 2015). While no systematic studies of CRISPR/Cas9 mutagenesis rates on any anopheline mosquitoes have been conducted, a few groups developing gene drive-related technologies (Adelman *et al.* 2017; Akbari *et al.* 2015; Champer *et al.* 2016; Marshall *et al.* 2015; Marshall *et al.* 2017) have recently reported high rates of mutagenesis when gRNAs and Cas9 were directly integrated into the genome of two *Anopheles* species (Gantz *et al.* 2015; Galizi *et al.* 2016; Hammond *et al.* 2016).

In summary, we report remarkably high rates of survival and mutagenesis in three different *Anopheles* species co-injected with Cas9 protein and sgRNAs targeting the *white* gene. Importantly, we describe the first, successful genetic engineering of *A. funestus*, demonstrating that the CRISPR/Cas9 system may even be useful in species where previous genome editing techniques proved too inefficient for practical use. Additionally, since high concentrations of sgRNA and Cas9 result in biallelic mutations, stable mutant lines can be rapidly generated, even when no visible marker is present. The following procedure can be used to generate such lines: (1) inject sgRNA and Cas9 into mixed sex eggs, (2) cross G0 survivors *en masse*, (3) isolate G0 females into individual ovicups, (4) screen five G1 larvae from each family for mutations (sequencing, T7EI, or an alternative assay), (5) conduct full sibling mating of families in which all G1 larvae are mutants, and (6) confirm stable generation of a mutant line by sequencing of G2 mosquitoes. The ability to rapidly and consistently create stable knockout lines should greatly accelerate mechanistic research into key cellular and molecular pathways in malaria mosquitoes.

We note that cleavage efficiency of the sgRNA/Cas9 complex is target site-dependent. In mammalian systems, it has been reported that the chromatin environment around the target site and certain features of the sgRNA sequence are major factors affecting the efficiency of DSB generation (Doench *et al.* 2014; Kuscu *et al.* 2014; Wang *et al.* 2015). Due to the limited number of sgRNAs we tested, we are unable to confirm whether these observations can be extended into *Anopheles*. However, the complete failure of AasgRNA2 to cause knockout is likely due to low thermodynamic stability of the sgRNA/Cas9 complex, or secondary structure at the target site preventing binding (Bassett and Liu 2014; Moreno-Mateos *et al.* 2015).

Having demonstrated the utility of the CRISPR/Cas9 system for site-specific mutagenesis of *Anopheles*, a logical next step is to systematically determine the efficiency of the system for integrating variously sized constructs into anopheline genomes via HDR. The ability to conduct efficient deletion and addition of known sequences at specific genomic positions will greatly speed progress toward genetic methods, such as gene drive, for the control of malaria vectors.

## Supplementary Material

Supplemental material is available online at www.g3journal.org/lookup/suppl/doi:10.1534/g3.117.1134/-/DC1.
