# Supplementary material for: Highly Efficient Site-Specific Mutagenesis in Malaria Mosquitoes Using CRISPR
Source: G3 (Bethesda). 2017 Dec 11;8(2):653–8. doi: 10.1534/g3.117.1134 (PMC5919725; doi:10.1534/g3.117.1134)
Supplement: Supplementary file 1 [file 653FileS1.docx]

**SUPPLEMENTAL FIGURES**


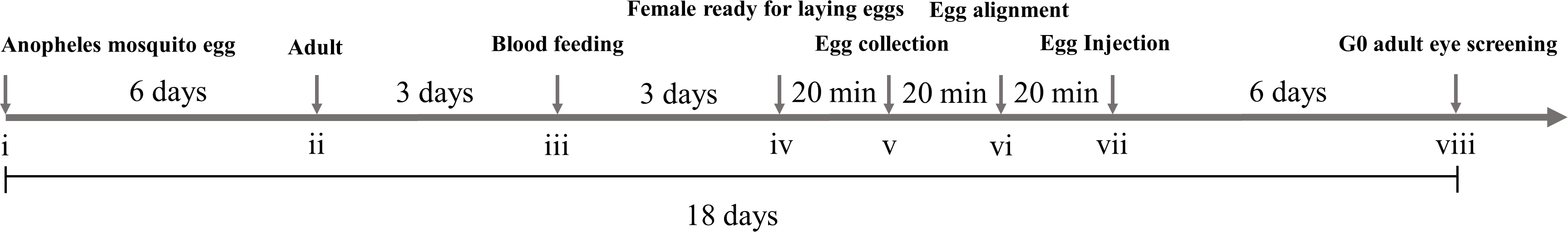


**Figure S1. Schematic of *Anopheles* mosquito embryo collection and CRISPR/Cas9**

**microinjections.**  *Anopheles* takes 12 days mature from egg to adult (i), Fresh *Anopheles* embryos were collected (ii), aligned (iii), and injected with CRISPR/Cas9 components (v) Injected embryos were then gently put into the water for development (6 days), and emerged G0 adults were subsequently screened for CRISPR/Cas9 induced mutations in target gene (vi). This entire procedure takes roughly 18 days to complete.

**
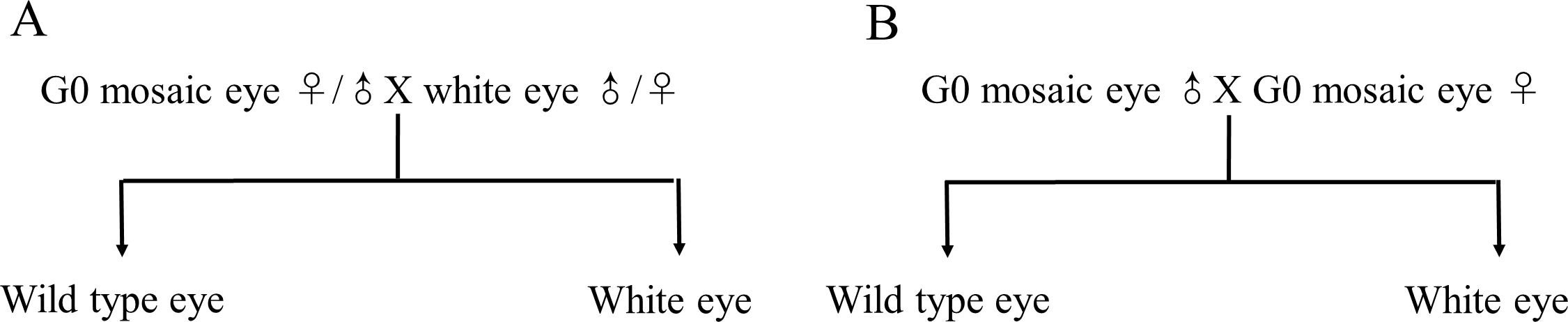
**

**Figure S2. Anopheles mosquito crossing strategies.** (A) Anopheles *coluzzii* mutant G0 cross with white-eye mutant line (M2). (B) Anopheles *albimanus* and Anopheles *funestus* mutant G0’s were inbred.


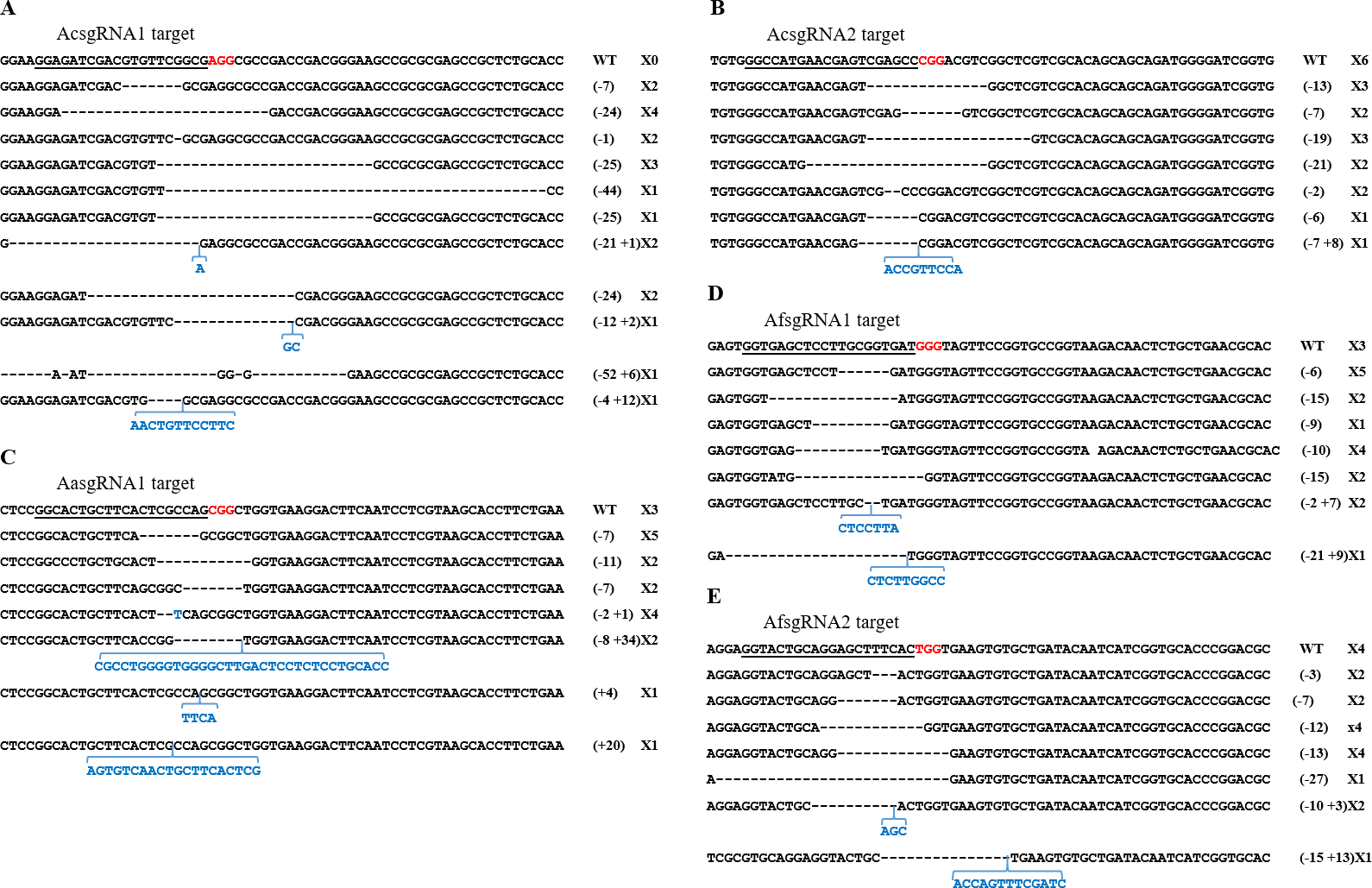


**Figure S3. Repair of CRISPR-induced double strand breaks results in a variety of indels.**  Sequencing of cloned PCR products from G_0_ injected mosquitoes possessing mosaic eyes revealed a variety of insertions and deletions adjacent to the guide target site. For each sgRNA, top line represents WT sequence; PAM sequences (NGG) are indicated in red, and gene disruptions resulting from insertions/deletions are indicated in blue/dash.


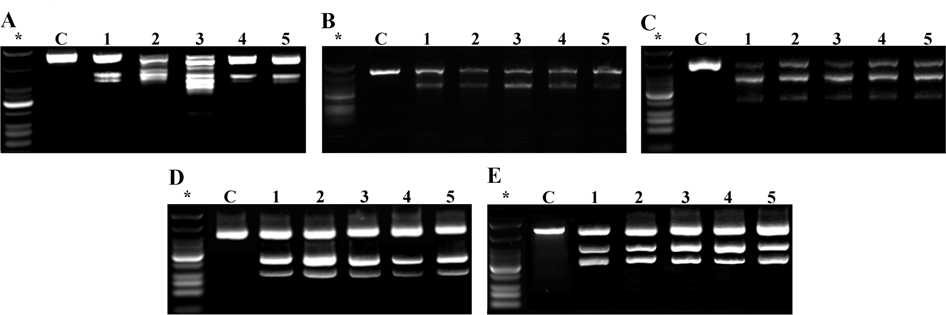


**Figure S4. The T7 Endonuclease assay can be used for rapid detection of CRISPR-generated mutant alleles.** For each successful guide RNA (A, AcsgRNA1; B, AcsgRNA2; C, AasgRNA1; D, AfsgRNA1; E, AfsgRNA2), PCR products from non-mosaic (C) and mosaic (1-5) mosquitoes were digested with T7 endonuclease. In all mosaic mosquitoes, partial digestion of the PCR product is evident, while in non-mosaic mosquitoes no digestion is visible.


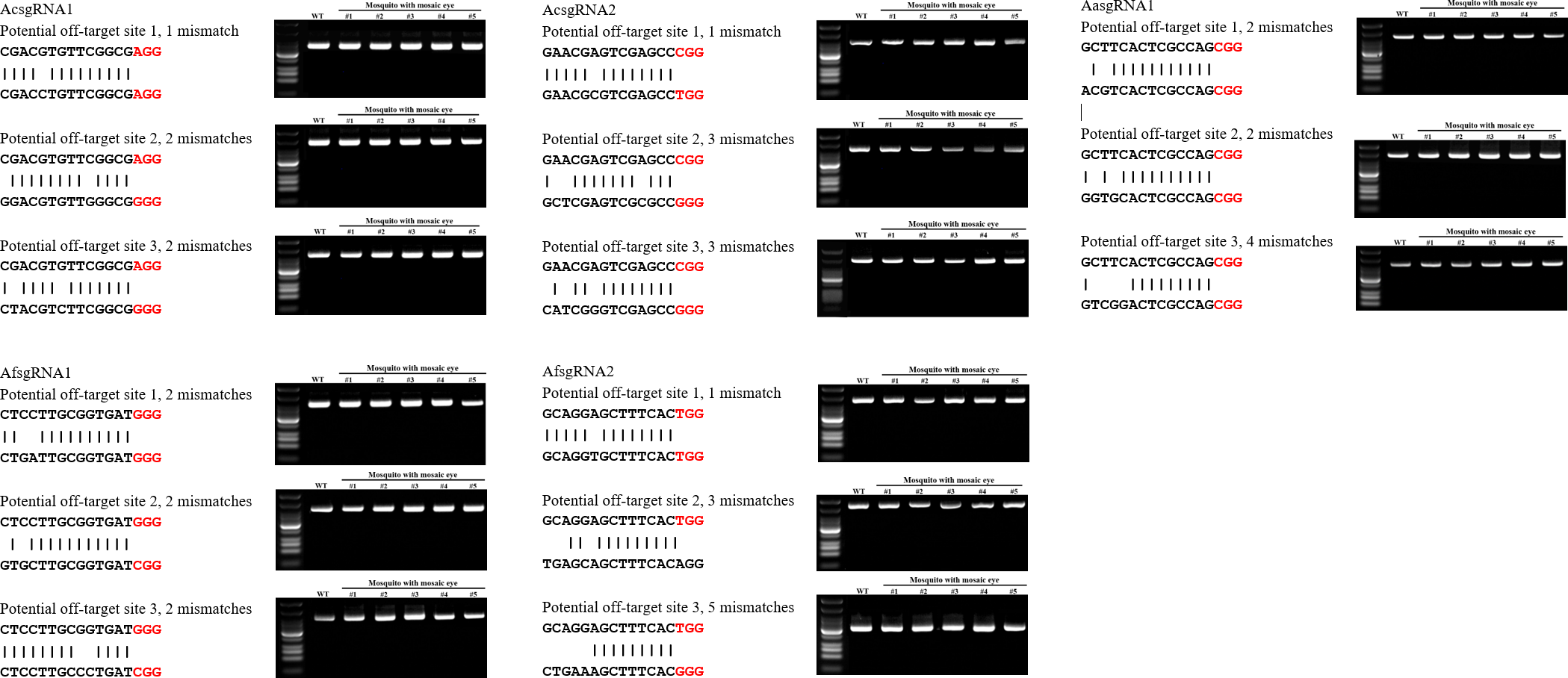


**Figure S5. No evidence for off-target mutagenesis of sgRNAs.** Three potential off target sites for each sgRNA were screened for mutagenesis activity by T7 endonuclease assay. PAM distal region sequence alignment of target locus and potential off-target loci. The potential off-target sites of sgRNAs in different *Anopheles* mosquitoes are indicated, and the PAM sites are labeled in red. T7 Endonuclease I (T7E1) assay of potential off-target loci. “WT” represented wild type mosquito, number from 1 to 5 indicated 5 different mosquitos with mosaic eye phenotype. No digestion is visible in any of the lanes.

Table S1. Primers used in this study.

| Species | Primer name | Primer sequence (5'-3') |
| --- | --- | --- |
| *A. coluzzii* | sgRNAF1 | GAAATTAATACGACTCACTATAGGAGATCGACGTGTTCGGCGGTTTTAGAGCTAGAAATAGC |
|  | PCRF1 | TGGAAGCTTGCTTGGGATAG |
|  | PCRR1 | GTCAGCGACGGTATGAACAG |
|  | sgRNAF1off-targetF1 | AACCACTGTCTGCTGAACTC |
|  | sgRNAF1off-targetR1 | GACAGGAACTCGCCCAAATA |
|  | sgRNAF1off-targetF2 | TCTTCCAGACGTACCTCGAT |
|  | sgRNAF1off-targetR2 | GGTGCTCGTTGAAGCTGTA |
|  | sgRNAF1off-targetF3 | TCCTCGAGCACGCAAATATC |
|  | sgRNAF1off-targetR3 | CCCGTTCTGGAAGTTGTACTC |
|  | sgRNAF2 | GAAATTAATACGACTCACTATAGGCCATGAACGAGTCGAGCCGTTTTAGAGCTAGAAATAGC |
|  | PCRF2 | AAACCTGCCCAACACCAT |
|  | PCRR2 | TAAGAGCCTGAGAGCCTGAC |
|  | sgRNAF2off-targetF1 | CACACCCAGAGCACATACAA |
|  | sgRNAF2off-targetR1 | GATCAGCGCGACGATATTCA |
|  | sgRNAF2off-targetF2 | GGCGATATGAAGCTCAAGGTA |
|  | sgRNAF2off-targetR2 | AAGGGATGACTGTCAATGGG |
|  | sgRNAF2off-targetF3 | CAGGTAGAGCGTGTTGATGTT |
|  | sgRNAF2off-targetR3 | GTTGTCCTCTCTAGAAGGGTTTATT |
| *A.albimanus* | sgRNAF1 | GAAATTAATACGACTCACTATAGGCACTGCTTCACTCGCCAGGTTTTAGAGCTAGAAATAGC |
|  | PCRF1 | AAGAGTTACGGTTCGGTGAAG |
|  | PCRR1 | AACATCAGGTGCTCCTTGG |
|  | sgRNAF1off-targetF1 | TCCACATACAGAATCCACTCAAC |
|  | sgRNAF1off-targetR1 | GTTCGGTCTCTTCTTGCTTCA |
|  | sgRNAF1off-targetF2 | GGCCGAGCAAAGTCAACA |
|  | sgRNAF1off-targetR2 | TGCTGAGCTACATTACGGTTTC |
|  | sgRNAF1off-targetF3 | TCTCAATCTACACACGGGAAAC |
|  | sgRNAF1off-targetR3 | ATGGATCTGGCGATCAAGAAG |
|  | sgRNAF2 | GAAATTAATACGACTCACTATAGGCTAGCGCGTTCAGCAGCGGTTTTAGAGCTAGAAATAGC |
|  | PCRF2 | GACAAGGCCACGCTGATAC |
|  | PCRR2 | TCCTGCAGTACCTCGTCAA |
| *A. funestus* | sgRNAF1 | GAAATTAATACGACTCACTATAGGTGAGCTCCTTGCGGTGATGTTTTAGAGCTAGAAATAGC |
|  | PCRF1 | GAAGAGCTACGGTTCGGTTAAG |
|  | PCRR1 | GGCTGGTGTATGGTGAGTATG |
|  | sgRNAF1off-targetF1 | CGGTGAATGTGTGCAGTTTG |
|  | sgRNAF1off-targetR1 | CGCTATTATAGTGGCACTGAGG |
|  | sgRNAF1off-targetF2 | GTCAATCTTCTCCTCGGATAGTG |
|  | sgRNAF1off-targetR2 | GCGAGATTCGACGTTTGATAAAG |
|  | sgRNAF1off-targetF3 | GTCAATCTTCTCCTCGGATAGTG |
|  | sgRNAF1off-targetR3 | GCGAGATTCGACGTTTGATAAAG |
|  | sgRNAF2 | GAAATTAATACGACTCACTATAGGTACTGCAGGAGCTTTCACGTTTTAGAGCTAGAAATAGC |
|  | PCRF2 | GAAGAGCTACGGTTCGGTTAAG |
|  | PCRR2 | GGCTGGTGTATGGTGAGTATG |
|  | sgRNAF2off-targetF1 | CGATGAGCGAAGAGTCCAAA |
|  | sgRNAF2off-targetR1 | TACCCAGCTGTGAAGCAATC |
|  | sgRNAF2off-targetF2 | CGTGTGGTCGCTTGGAATAA |
|  | sgRNAF2off-targetR2 | CGCTGGTAAGTTGGTGGTAAA |
|  | sgRNAF2off-targetF3 | CCTACCGCCAGGAATGTAATG |
|  | sgRNAF2off-targetR3 | GGACCGAGGCTGAAATGTTAT |
|  | Universal-sgRNAR | AAAAGCACCGACTCGGTGCCACTTTTTCAAGTTGATAACGGACTAGCCTTATTTTAACTTGCTATTTCTAGCTCTAAAAC |

Table S2 *In silico*-prediction of microhomology-associated DNA repair

| sgRNA | Target | Deletion length^%^ | Microhomology score^&^ |
| --- | --- | --- | --- |
| AcsgRNA1 | GGAGATCGACGTGTTCGGCG | 3 - 53 | 5878.3 |
| AcsgRNA2 | GGCCATGAACGAGTCGAGCC | 6 - 57 | 5654.9 |
| AasgRNA1 | GGCACTGCTTCACTCGCCAG | 4 - 51 | 5177.2 |
| AfsgRNA1 | GGTGAGCTCCTTGCGGTGAT | 6 - 53 | 5028.7 |
| AfsgRNA2 | GGTACTGCAGGAGCTTTCAC | 6 - 54 | 3755.8 |

^%^ *In silico* (<http://www.rgenome.net/mich-calculator/>) predicted deletion length mediated by CRISPR/Cas9

^&^ The sum of pattern scores, which associated with microhomology deletion patterns (Bae. et al, 2014)

Bae, S. J Kweon, H. S. Kim and J.S. Kim, 2014 Microhomology-based choice of Cas9 nuclease target sites. Nat. Med. 11: 705-706
